# Aplastic Anemia With Non-Occlusive Mesenteric Ischemia

**DOI:** 10.7759/cureus.72727

**Published:** 2024-10-30

**Authors:** Rithvik Rai, Abiola Kehinde

**Affiliations:** 1 Geriatrics and Endocrinology, Hinchingbrooke Hospital, Huntingdon, GBR; 2 Endocrinology and Diabetes, Hinchingbrooke Hospital, Huntingdon, GBR

**Keywords:** aplastic crisis, ischemic colitis, mesenteric ischaemia, severe hematemesis, staph aureus colitis

## Abstract

This case highlights the multifaceted challenges of managing aplastic anemia, especially when complicated by non-occlusive mesenteric ischemia (NOMI). The patient’s clinical course underscores the importance of a conservative, multidisciplinary approach in balancing the risks of invasive procedures with the need for effective diagnostics and treatment. Recognizing the risks associated with pancytopenia, including life-threatening bleeding and infections, is critical in managing such patients. Careful monitoring and timely interventions, particularly in an intensive care setting, are essential to improve outcomes in complex cases like this one.

## Introduction

Aplastic anemia is a rare hematological disorder characterized by bone marrow failure, leading to pancytopenia. Patients typically present with complications such as bleeding due to thrombocytopenia, infections from neutropenia, and fatigue from anemia [1]. In this case, the patient’s management was further complicated by non-occlusive mesenteric ischemia (NOMI).

This case underscores the complexities involved in managing aplastic anemia, particularly when complicated by NOMI. The concurrent presence of thrombocytopenia, neutropenia, and abdominal complications presents significant challenges for clinical decision-making. The decision to delay invasive procedures such as endoscopy was driven by the necessity to stabilize the patient’s thrombocytopenia first. Management of NOMI typically involves conservative measures, as surgical intervention is often not required [3].

Key learning points from this case include the intricate management required for patients with aplastic anemia, particularly when compounded by conditions like NOMI, highlighting the importance of multidisciplinary collaboration. Furthermore, it emphasizes the heightened risk of complications, such as bleeding and infections, faced by patients with pancytopenia. The conservative management approach adopted in this case, including the postponement of invasive diagnostics until platelet levels were stabilized, exemplifies the need to balance the risks associated with thrombocytopenia against the urgency for diagnosis. Lastly, the case illustrates the critical importance of close monitoring and hemodynamic stability in the intensive care setting to promptly identify any deterioration in the patient’s condition.

## Case presentation

**Figure 1:**
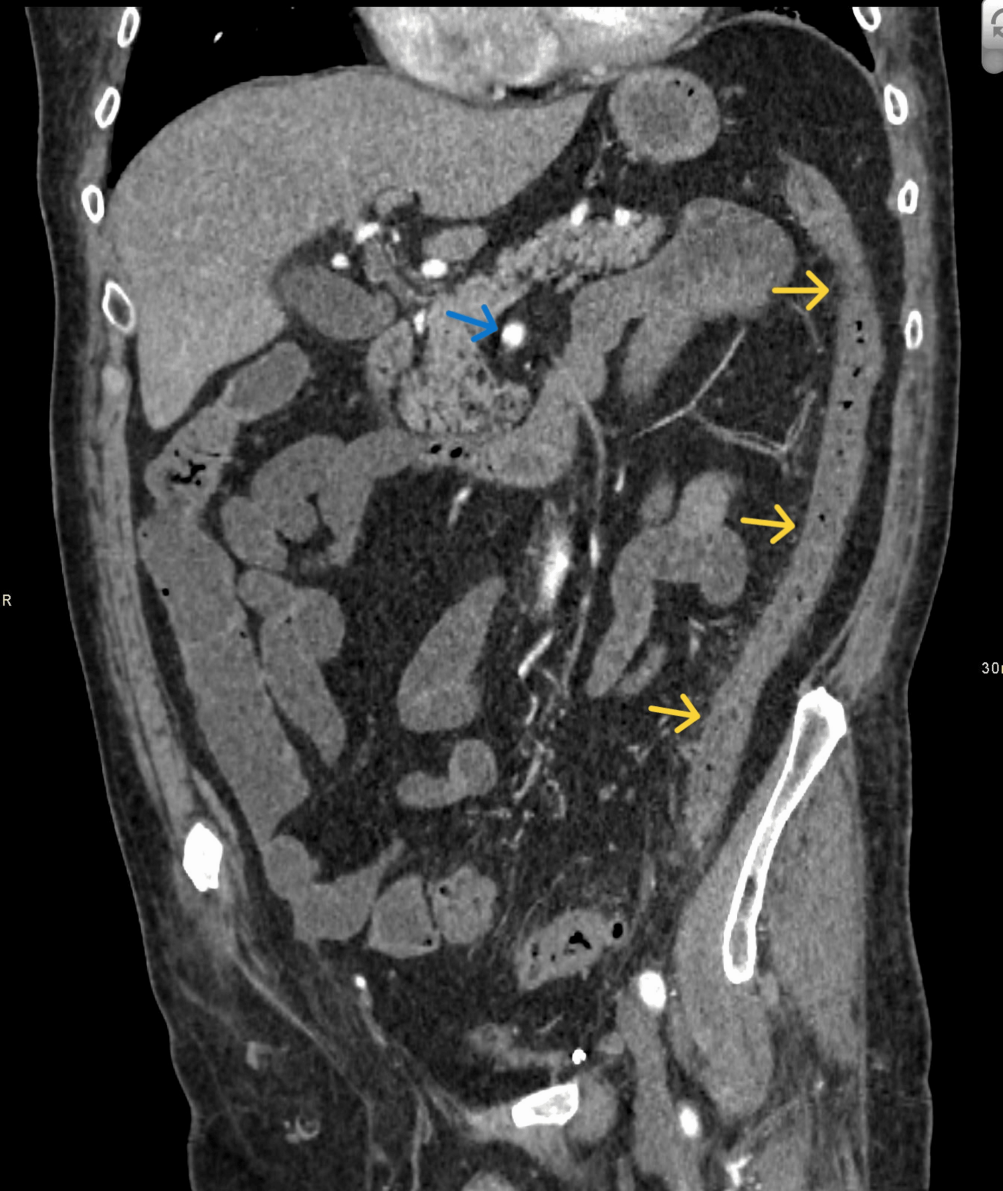
Selected coronal reconstruction image of a CT abdomen and pelvis study in the arterial phase demonstrating 'circumferential wall and fat stranding' (yellow arrows). Note that the superior mesenteric artery (SMA) is patent and shows homogeneous opacification (blue arrow). Blue Arrow: Superior mesenteric artery Yellow Arrows: Inflamed left colon. Blue Arrow: Superior mesenteric artery Yellow Arrows: Inflamed left colon.

A patient in their seventies presented to the emergency department with two months of worsening lower abdominal pain. They experienced three episodes of hematemesis and reported dark stools, although no visible blood was noted. A CT scan revealed features consistent with colitis and possible NOMI (Figure 1).

The patient's medical history included aplastic anemia, hypertension, a factor V Leiden mutation, and previous thrombotic events, including a pulmonary embolism in 2007 and cerebral venous thrombosis in 2003. They also had a history of bladder stones and hemorrhoid surgery.

On examination, the patient was alert but in distress, with generalized abdominal tenderness, particularly in the suprapubic area, without guarding or distension. There was no pedal edema, and bowel sounds were sluggish. 

Laboratory results showed a hemoglobin level of 84 g/L, platelet count of 7 x 10^9^/L, and white cell count of 0.9 x 10^9^/L. Blood cultures were positive for Staphylococcus aureus, and a venous blood gas analysis indicated a pH of 7.44 with a lactate level of 6.3 mmol/L. The CT confirmed left-sided colitis with segmental involvement and patent mesenteric arteries (Figure 1).

The differential diagnosis included NOMI, diverticulitis, and inflammatory bowel disease, with the latter being less likely. The patient was managed conservatively with intravenous fluids, antibiotics (ciprofloxacin and metronidazole), and platelet transfusions. Due to thrombocytopenia and ongoing hematemesis, endoscopy was deferred. The hematology team recommended GCSF once hemodynamic stability was achieved.

Despite resuscitation efforts, the patient continued to experience hypotension and was transferred to the intensive care unit for close monitoring. Although there was a temporary improvement in blood pressure, the patient’s condition remained guarded.

## Discussion

This case highlights the complexities associated with managing aplastic anemia, particularly when further complicated by NOMI. The interplay of thrombocytopenia, neutropenia, and abdominal complications presents significant challenges in treatment. In this case, the decision to postpone invasive procedures such as endoscopy was primarily driven by the urgent need to correct severe thrombocytopenia to mitigate the risk of bleeding. Generally, the management of NOMI favors conservative measures, with surgical intervention typically reserved for cases of clinical deterioration or failure of conservative management [4].

Management of NOMI typically leans toward conservative measures, as surgical intervention is usually warranted only in cases with definitive evidence of bowel necrosis or perforation [4]. Imaging techniques, particularly CT scans, play a vital role in the early diagnosis of NOMI, facilitating the prompt initiation of appropriate therapeutic measures [5]. Conservative management strategies often encompass fluid resuscitation, antibiotic therapy, and platelet transfusions aimed at rectifying the underlying hematological abnormalities [6].

The risk of bleeding in patients with aplastic anemia is considerably elevated due to the profound thrombocytopenia inherent to the condition. This significantly complicates the decision-making process for diagnostic procedures like endoscopy, which can exacerbate bleeding in patients with already impaired hemostasis. In this case, the critical collaboration among hematologists, gastroenterologists, and intensivists was essential for balancing the risks and benefits of invasive diagnostics versus conservative management.

NOMI is primarily associated with systemic hypoperfusion and vasospasm rather than thrombotic occlusion of the mesenteric arteries [7]. Current literature underscores the importance of early recognition and conservative management of NOMI, reserving surgical interventions for patients exhibiting deteriorating clinical status or clear signs of bowel infarction. This reinforces the necessity for a multidisciplinary approach to patient care, ensuring that all clinical aspects are addressed comprehensively [8].

## Conclusions

This case illustrates the complex challenges involved in managing a patient with aplastic anemia, particularly when further complicated by NOMI. The patient's severe pancytopenia, coupled with gastrointestinal bleeding, necessitated a cautious, conservative approach to treatment. In conditions where invasive procedures like endoscopy carry significant risks of bleeding, it becomes crucial to stabilize the patient's hematological parameters first. The decision to delay endoscopy in favor of supportive care, such as fluid resuscitation, antibiotic therapy, and transfusions, highlights the importance of individualizing treatment plans based on the patient's overall clinical stability.

Managing NOMI in the context of severe pancytopenia requires close multidisciplinary collaboration. In this case, the involvement of hematologists, gastroenterologists, and intensivists was vital to balance the risks and benefits of conservative versus invasive approaches. Early recognition of NOMI via imaging allowed the medical team to initiate conservative measures promptly, thereby potentially preventing progression to bowel infarction, which would have required surgical intervention and further complicated the patient’s fragile state.

Ultimately, this case underscores the importance of vigilant monitoring in patients with aplastic anemia and complicated comorbidities. Intensive care settings allow for the necessary close hemodynamic monitoring and timely interventions, which are critical for optimizing outcomes. The delicate balance between intervention and conservative management, tailored to the patient's condition, was key in addressing both the underlying hematological disorder and the gastrointestinal complication.
